# Phylogeny of *Vibrio vulnificus* from the Analysis of the Core-Genome: Implications for Intra-Species Taxonomy

**DOI:** 10.3389/fmicb.2017.02613

**Published:** 2018-01-05

**Authors:** Francisco J. Roig, Fernando González-Candelas, Eva Sanjuán, Belén Fouz, Edward J. Feil, Carlos Llorens, Craig Baker-Austin, James D. Oliver, Yael Danin-Poleg, Cynthia J. Gibas, Yechezkel Kashi, Paul A. Gulig, Shatavia S. Morrison, Carmen Amaro

**Affiliations:** ^1^Estructura de Investigación Interdisciplinar en Biotecnología y Biomedicina BIOTECMED, University of Valencia, Valencia, Spain; ^2^Departmento de Microbiología y Ecología, Universidad de Valencia, Valencia, Spain; ^3^Biotechvana, Parc Cientific, Universitat de Valencia, Valencia, Spain; ^4^Joint Research Unit on Infection and Public Health FISABIO-Salud Pública and Universitat de Valencia-I2SysBio, Valencia, Spain; ^5^CIBEResp, National Network Center for Research on Epidemiology and Public Health, Instituto de Salud Carlos III, Valencia, Spain; ^6^Department of Biology and Biochemistry, University of Bath, Bath, United Kingdom; ^7^Centre for Environment, Fisheries and Aquaculture Science, Weymouth, United Kingdom; ^8^Department of Biological Sciences, University of North Carolina at Charlotte, Charlotte, NC, United States; ^9^Duke University Marine Lab, Beaufort, NC, United States; ^10^Faculty of Biotechnology and Food Engineering, Technion–Israel Institute of Technology, Haifa, Israel; ^11^Department of Bioinformatics and Genomics, the University of North Carolina at Charlotte, Charlotte, NC, United States; ^12^Department of Molecular Genetics and Microbiology, University of Florida, Gainesville, FL, United States

**Keywords:** microbial evolution, pathogens, SNP, *Vibrio vulnificus*, core genome, virulence plasmid, pathovar, biotype

## Abstract

*Vibrio vulnificus* (Vv) is a multi-host pathogenic species currently subdivided into three biotypes (Bts). The three Bts are human-pathogens, but only Bt2 is also a fish-pathogen, an ability that is conferred by a transferable virulence-plasmid (pVvbt2). Here we present a phylogenomic analysis from the core genome of 80 Vv strains belonging to the three Bts recovered from a wide range of geographical and ecological sources. We have identified five well-supported phylogenetic groups or lineages (L). L1 comprises a mixture of clinical and environmental Bt1 strains, most of them involved in human clinical cases related to raw seafood ingestion. L2 is formed by a mixture of Bt1 and Bt2 strains from various sources, including diseased fish, and is related to the aquaculture industry. L3 is also linked to the aquaculture industry and includes Bt3 strains exclusively, mostly related to wound infections or secondary septicemia after farmed-fish handling. Lastly, L4 and L5 include a few strains of Bt1 associated with specific geographical areas. The phylogenetic trees for ChrI and II are not congruent to one another, which suggests that inter- and/or intra-chromosomal rearrangements have been produced along Vv evolution. Further, the phylogenetic trees for each chromosome and the virulence plasmid were also not congruent, which also suggests that pVvbt2 has been acquired independently by different clones, probably in fish farms. From all these clones, the one with zoonotic capabilities (Bt2-Serovar E) has successfully spread worldwide. Based on these results, we propose a new updated classification of the species based on phylogenetic lineages rather than on Bts, as well as the inclusion of all Bt2 strains in a pathovar with the particular ability to cause fish vibriosis, for which we suggest the name “piscis.”

## Introduction

*Vibrio vulnificus* is an emerging zoonotic pathogen that inhabits brackish water ecosystems from temperate and tropical areas and whose geographical distribution has recently extended to Northern countries due to global warming (Baker-Austin et al., 2013, 2017). The pathogen survives in water, either associated with the mucous surfaces of algae and aquatic animals or as a free-living bacterium that can be concentrated by filtering organisms such as oysters (Oliver, 2015).

*V. vulnificus* was defined as a bacterial species in 1976 (Farmer, 1979) and was later split into three biotypes (Bt) on the basis of differences in genotypic and phenotypic traits as well as in host range (Tison et al., 1982; Bisharat et al., 1999). The three Bts are opportunistic human pathogens, but Bt2 is also pathogenic for aquatic animals. The zoonotic strains belong to the same serovar (Ser) and are classified as Bt2-SerE (Biosca et al., 1997).

The various diseases caused by this species are known as vibriosis. Human vibriosis presents as two main forms depending on the pathogen's route of entry into the body, ingestion or contact (Oliver, 2015). In the first case, the pathogen is ingested with raw seafood, colonizes the intestine and causes gastroenteritis and/or primary septicemia. In the second case, the pathogen crosses the skin barrier during an injury or directly colonizes a preexisting wound causing local but severe necrosis and/or secondary septicemia. The main factor that predisposes to death by sepsis is a high level of serum iron as a consequence of multiple pathologies (e.g., hemochromatosis, diabetes, cirrhosis, and viral hepatitis) (Oliver, 2015).

Epidemiological data on human vibriosis from the Centers for Disease Control and Prevention (CDC) estimate that around 80,000 people are infected by *Vibrio* spp. each year in the USA and that, of these, *V. vulnificus* is responsible for most of the fatal cases (case fatality rate >50% for septicemia; Jones and Oliver, 2009). Thus, *V. vulnificus* is responsible for over 95% of seafood-related deaths (Jones and Oliver, 2009), the highest fatality rate of any known food-borne pathogen (Rippey, 1994). In addition, increasing incidents of infections are occurring globally, with cases reported in Europe and Asia (Hlady and Klontz, 1996; Baker-Austin et al., 2013; Lee et al., 2013b). Crucially, infections currently appear to be increasing in both the USA and in Europe (Newton et al., 2012; Baker-Austin et al., 2013).

Regarding fish vibriosis, eels seem to be the most susceptible host, especially under farming conditions (Amaro et al., 2015). The pathogen colonizes the eel gills, enters the blood and causes death by septicemia, even in healthy individuals (Marco-Noales et al., 2001). Eel vibriosis occurs in farms as epizootics or outbreaks of high mortality that can lead to the closure of the farm if the disease is not controlled promptly. Epizootiological data suggest that outbreaks of eel vibriosis have been registered in all of the countries where eels are cultured in brackish waters, including fish farms located in Northern-European countries (Haenen et al., 2013).

The genetic basis for human virulence is only partially known, although most studies suggest that all strains of the species may have the ability to infect humans regardless of their origin, clinical, or environmental (Gulig et al., 2005). In contrast, the ability to infect fish is dependent on a virulence plasmid (pVvBt2) that is only present in Bt2 strains (Lee et al., 2008; Roig and Amaro, 2009). The plasmid encodes resistance to the innate immunity of eels, and probably other teleost (Lee et al., 2008, 2013a; Pajuelo et al., 2015). Interestingly, pVvbt2 can be transmitted among bacteria aided by a second, conjugative plasmid, which is widespread in the species (Lee et al., 2008).

The aim of this study is to describe the phylogenetic groups within the species and compare them with the current intraspecific groups from the characterization of the core genome of species (CGS), the core genome of plasmid pVvbt2 (CGP) and the core genome of human virulence-related genes (CGV). Our results highlight the importance of aquaculture industry in the recent evolution and epidemic spread of the species and support the intra-specific classification in lineages instead of Bts, as well as the inclusion of a pathovar grouping all fish virulent isolates for which we propose the name “piscis.”

## Materials and methods

### Bacterial isolates, culture conditions, DNA extraction, and sequencing

The genomes used in this study and the main features of the corresponding strains are detailed in Table 1. The strains whose genome was sequenced are marked in Table 1. These strains were routinely grown in Tryptone Soy Broth or agar plus 5 g/l NaCl (TSB-1 or TSA-1, Pronadisa, Spain) at 28°C for 24 h. The strains were maintained both as lyophilized stocks and as frozen stocks at −80°C in marine broth (Difco) plus 20% (v/v) glycerol.

**Table 1:** Origin, year of isolation, biotype, serovar, virulence-related typing, and genome accession number of *V. vulnificus* strains used in this study.

**Strain**	**Origin**	**Country**	**Isolation Year**	**Biotipe Serovar Clade^a^**	**vvpdh^b^**	**vcg^b^**	**Sequence accession**
32	Human blood	Israel	1997?	3/O	+	E	SAMN07739065
162	Human blood	Israel	1997	3/O	+	E	SAMN07739066
2322	Fish pound water	Israel	1997	1	–	E	GCA_000743165.1
491771	Human blood	Israel	1997	3	+	E	GCA_000743115.1
106-2A^*^;^***^(V Harwood lab)	Oyster	U.S.A.	<2011	1	–	C	SAMN07739068
11028^***^	Human wound	Israel	1997	3/O	+	E	GCA_002074875.1
12^***^	Health tilapia	Israel	2002	3/O	+	E	GCA_002074885.1
93U204	Diseased tilapia	Taiwan	No reported	1	+	C	GCA_000746665.1
94385^*^ (C Amaro lab)	Human wound	Spain	2001	1	+	E	SAMN07739067
94-8-112^*^,^**^,^***^ (C Amaro lab)	Human wound	Denmark	1994	2/E	+	E	SAMN07739076
94-9-119^*^ (C Amaro lab)	Human wound	Denmark	1994	1	+	E	SAMN07739069
95-8-161^*^,^**^ (C Amaro lab)	Diseased eel	Denmark	1995	2/I	+	E	SAMN07739070
95-8-6^*^,^**^ (C Amaro lab)	Diseased eel	Denmark	1995	2/I	–	E	SAMN07739071
95-8-7^*^,^**^ (C Amaro lab)	Diseased eel	Denmark	1995	2/I	+	E	SAMN07739072
960426-1_4C^*^,^**^	Diseased eel	Denmark	1996	2/NT	+	E	SAMN07739073
99-578_DP-B1	Oyster	U.S.A.	1998	1	–	C	GCA_000788325.1
99-796_DP-E7	Oyster	U.S.A.	1998	1	–	E	GCA_000788315.1
A14^*^,^**^ (C Amaro lab)	Diseased eel	Spain	2002	2/A	–	E	SAMN07739074
AB17-319^*^,^***^ (JD Oliver lab)	Oyster	U.S.A.	2005	1	–	C/E	SAMN07739075
ATCC_27562^T^ (type strain)	Human blood	U.S.A.	No reported	1	–	E	GCA_000299635.1
ATCC_29306	Human wound	U.S.A.	No reported	1	+	E	GCA_001471415.1
ATCC_29307	Human blood	U.S.A.	No reported	1	–	C	GCA_001471465.1
ATCC_33147	Diseased eel	Japan	1979	2/E	+	E	GCA_000764895.1
ATCC_43382	Human blood	U.S.A.	No reported	1	–	E	GCA_001471305.1
ATL_6-1306	Human blood	U.S.A.	1996	1	+	C	GCA_000788335.1
ATL_71503	Human blood	U.S.A.	1996	1	–	E	GCA_000788345.1
B2	Human blood	China	2010	1	–	E	GCA_000303175.1
BAA87	Human wound	Israel	1996	3	+	E	GCA_000576265.1
C7184 (or CDC7184)^*^,^***^	Human blood	U.S.A.	1977	1	+	C	SAMN07739077
CECT4604^*^,^**^,^***^ (C Amaro lab)	Diseased eel	Spain	1990	2/E	+	E	SAMN07739078
CECT4606 ^*^,^***^ (C Amaro lab)	Healthy eel	Spain	1990	1	+	E	LAXL00000000
CECT4608^*^ (C Amaro lab)	Eel tank water	Spain	1990	1	+	C	SAMN07739079
CECT4865^*^,^**^ (C Amaro lab)	Diseased shrimp	Taiwan	No reported	2/E	+	E	SAMN07739080
CECT4866^*^,^**^ (C Amaro lab)	Human blood	Australia	1997	2/E	+	E	LABE00000000
CECT4999^*^, ^**^ (C Amaro lab)	Diseased eel	Spain	1999	2/E	+	E	GCA_002215135.1
CECT5763^*^,^**^,^***^ (C Amaro lab)	Eel tank water	Spain	2002	2/E	+	E	LEAM00000000
CECT5769^*^,^**^,^***^ (C Amaro lab)	Diseased eel	Spain	2004	2/A	–	E	LABF00000000
CECT7030^*^,^**^ (C Amaro lab)	Diseased eel	Denmark	2004	2/A	–	E	SAMN07739081
CECT898^*^,^**^ (C Amaro lab)	Diseased eel	Japan	1979	2/E	+	E	SAMN07739082
CG100^*^ (C Amaro lab)	Oyster	Taiwan	1993	1	+	C	SAMN07739083
CG64	Oyster	Taiwan	1993	1	+	C	GCA_000959775.1
CIP8190^*^, ^**^,^***^ (C Amaro lab)	Human blood	France	1980	2/E	+	E	LAXM00000000
CMCP6^***^	Human blood	South Korea	<2003	1	+	C	GCA_000039765.1
E64MW	Human wound	No reported	No reported	1	+	E	GCA_000269745.1
ENV1^*^,^***^ (JD Oliver lab)	Oyster	U.S.A.	2005	1	–	E	SAMN07739084
FLA112 (ATL9824)^*^,^***^ (A dePaola lab)	Human blood	U.S.A.	1994	1	+	C	SAMN07739085
FLA144 (CDC 90-3095 or ORL 1506)^*^,^***^ (A dePaola lab)	Human blood	U.S.A.	1995	1	–	E	SAMN07739086
FORC_009	Stool sample	South Korea	2008	1	–	C	GCA_001433435.1
FORC_016	Human blood	South Korea	2009	1	–	C	GCA_001653775.1
FORC_017	Human blood	South Korea	2014	1	+	C	GCA_001675245.1
JY1305	Oyster	U.S.A.	1999	1	–	E	GCA_000269725.1
JY1701	Oyster	U.S.A.	1999	1	+	E	GCA_000269765.1
LSU1015^*^,^***^ (JD Oliver lab)	Human wound	U.S.A.	<1998	1	–	C	PRJNA279176
LSU1657^*^,^***^ (JD Oliver lab)	Human wound	U.S.A.	<1998	1	+	E	SAMN07739088
LSU2098^*^,^***^ (JD Oliver lab)	Human wound	U.S.A.	<1998	1	–	E	SAMN07739089
MO6-24/O^***^	Human blood	South Korea	1986	1	+	C	GCA_000186585.1
NB-VV-101	Tilapia	Israel	1997	1	–		GCA_000743155.1
NV1	Seawater	Taiwan	2011	1	+	C	GCA_000959755.1
NV22	Seawater	Taiwan	2011	1	–	C	GCA_000960125.1^c^
PD-2-51^*^,^**^,^***^ (C Amaro lab)	Seawater	Spain	2003	2/E	+	E	SAMN07739090^c^
R02^*^,^**^,^***^ (C Amaro lab)	Diseased eel	Spain	2002	2/E	–	E	SAMN07739091^c^
Ra3^*^,^**^ (C Amaro lab)	Diseased eel	Spain	2011	2/E	+	E	SAMN07739092^c^
Riu1^*^	Seawater	Spain	2003	1	–	E	SAMN07739093^c^
S2-22^*^,^***^ (V Harwood lab)	Water	U.S.A.	2004	1	+	C	SAMN07739094^c^
S3-16^*^,^***^ (V Harwood lab)	Water	U.S.A.	2005	1	+	C	SAMN07739095^c^
SC9613	Crab	South Korea	1996	1	–	E	GCA_000959745.1^c^
SC9629	Seafood	South Korea	1996	1	–	E	GCA_000967055.1^c^
SC9729	Seawater	South Korea	1997	1	–	E	GCA_000959825.1^c^
SC9740	Seawater	South Korea	1997	1	–	E	GCA_000959765.1^c^
SC9761	Oyster	South Korea	1997	1	–	E	GCA_000959835.1^c^
SC9794	Tidal mudflat	South Korea	1997	1	+	C	GCA_000959845.1^c^
SREL119^*^,^***^ (JD Oliver lab)	Sediment	U.S.A.	<2006	1	–	E	SAMN07739096^c^
SREL314^*^,^***^ (JD Oliver lab)	Water	U.S.A.	<2006	1	–	E	SAMN07739097^c^
SS108-A3A^*^,^***^ (JD Oliver lab)	Oyster	U.S.A.	2005	1	–	E	SAMN07739098^c^
V252	Human blood	Israel	2004	1/clade B	–	E	GCA_001277815.1^c^
VV4-03	Human blood	Israel	2003	3	+	E	GCA_000743095.1^c^
VV9-09	Human blood	Israel	1999	3	+	E	GCA_000743105.1
vvyb1^***^	Healthy Tilapia	Israel	2004	3/O	+	E	GCA_000342305.2^c^
yb158^***^	Healthy Tilapia	Israel	2005	1/clade A	+	C	GCA_001013325.1
YJ016 ^***^	Human blood	Taiwan	1993	1	+	C	GCA_000009745.1

* *Strains whose genomes were sequenced in this study. The laboratory that purchased the strain is indicated in parenthesis*.

** *Strains used for virulence plasmid analysis*.

*** *Strains used for Vibrio species analysis*.

a *O-antigen serovar was determined for Bt2 and 3 isolates according to Biosca et al. (1997): Clade A and B were described by Danin-Poleg et al. (2015) and Efimov et al. (2015), respectively. NT, non typable*.

b *vvpdh (V. vulnificus potentially dangerous for humans); pilF polymorphism associated with human virulence (Roig et al., 2010): vcg (virulence correlated gene: E, environmental type, C, clinical type, C/E, clinical and environmental type) (Rosche et al., 2005)*.

c *Waiting for definitive accession*.

DNA was extracted using GenElute™ Bacterial Genomic DNA (Sigma, Spain) from bacteria grown with shaking at 28°C for 12 h. Samples with a DNA concentration of 10–15 ng/μl were used for sequencing with Illumina Genome Analyzer technology GAII (Illumina MiSeq) flow cell in the Genome Analysis Centre in Norwich (UK) and the SCSIE of the University of Valencia (Spain). To this end, unique index-tagged libraries for each sample (up to 96 strains) were created using TruSeq DNA Sample Preparation for subsequent cluster generation (Illumina cBot), and up to 12 separate libraries were sequenced in each of eight channels in Illumina Genome Analyser GAII cells with 100-base paired-end reads. The index-tag sequence information was used for downstream processing to assign reads to the individual samples (Harris et al., 2010).

### Genomes selected as reference

The genomes of the Bt1 strain YJ016 (NC_005139 and NC_005140) and the plasmids pR99 (AM293858) and p4602-2 (AM293860) were used as templates for all the genomic analysis. The genome of the strain YJ016 was selected because it is one of the few genomes of *V. vulnificus* that has been accurately closed and annotated (Chen et al., 2003). The YJ016 genome contains 5,097 genes (3,387 in chromosome I and 1,710 in chromosome II), pR99 contains 71 genes and p4602-2 contains 67 genes.

### Genome sequence assembly

Reads for each genome were done using SPAdes version 3.6.1 (Bankevich et al., 2012) with kmers from 21 to 127 and the careful option to reduce mismatches and short indels. Then, multiple sequence alignments were obtained by using Progressive Mauve software with default options (Darling et al., 2004). Locally Collinear Blocks (LCBs) with a size larger than 1 kb that were present in all the genomes were used to define the core genomes. The selected LCBs were concatenated to be used in subsequent analyses to build a core genome multiple alignment.

### Core genome

We selected seven *Vibrio* species that had at least one fully sequenced and annotated genome to define the core genome of the genus (CGG). Table S1 summarizes the characteristics of the closed *Vibrio* genomes selected for the study.

The identification of each gene was performed using local BLAST searches (Wang et al., 2003). Three independent searches, one per chromosome and plasmid, were performed, and a database was generated for each genome. The resulting sequences were mapped onto reference genes using Geneious 6.1.6 (Biomatters) software. As the sequences used for the generation of the databases were not closed genomes (draft genomes/contigs), only the genes present in all the genomes, with a minimum length of 80% of the total length of the homologous gene in the reference genome and with a DNA identity higher than 70%, were included in the CGS, the CGP or the CGG for further analysis.

In order to refine the preliminary alignment for the entire core, individual alignments for each gene were performed using the program MAFFT (Katoh and Standley, 2013). The elimination of unaligned ends and genes that did not match the above requirements was performed using an *in-house* Python script. Once all the genes were aligned, a concatenated sequence was generated for the CGS, the CGP and the CGG.

### Functional analysis of the CGS

Functional annotation was performed using GPRO (Futami et al., 2011). Gene descriptions were obtained by blasting the predicted protein sequences against the NCBI non-redundant proteins (NR) and the Clusters of Orthologous Groups (COGs) databases (Tatusov, 2000) as reference subjects. Protein accessions obtained from the annotation based on NR were used to add additional Gene Ontology (GO) (Gene Ontology Consortium, 2008) and Enzyme Commission (EC) (Bairoch, 2000) designations. Information about metabolic pathways was retrieved via the web from the KEGG database (Kotera et al., 2012) based on EC numbers relation.

### Single nucleotide polymorphisms (SNPs) identification

SNPs were identified in the genomes from the coding regions as described previously (Harris et al., 2010) with appropriate SNP cutoffs to minimize the number of false-positive/negative calls. SNPs were filtered to remove those at sites with a SNP quality score lower than 40 and/or with read coverage below 25X in this region. SNPs at sites with heterogeneous mappings were also filtered out if the SNP was present in fewer than 85% of reads for that position.

### Virulence genes to define the human virulence-related core genome (VCGS)

We identified human virulence-related genes in the CGS from previously published data to define the human virulence-related core genome (VCGS) (Chakrabarti et al., 1999; Chen et al., 2004; Horstman et al., 2004; Gulig et al., 2005; Bogard and Oliver, 2007; Alice et al., 2008; Oh et al., 2008; Brown and Gulig, 2009; Liu et al., 2009; Lee et al., 2010; Chen and Chung, 2011; Kim et al., 2011).

### Phylogenetic reconstruction and congruence analysis

All the phylogenetic trees for CGG, CGS, CGP, and VCGS were reconstructed using the maximum-likelihood (ML) method with PhyML software (Guindon et al., 2009). The best evolutionary model for each dataset was determined with jModelTest (Posada, 2008) and considering the Akaike information criterion (AIC) (Akaike, 1974). The selected models were the generalized time-reversible model (GTR) with Gamma (+G) distribution and invariant positions (+I) (Tavaré, 1986) for SNP analysis. The pVvBt2 phylogeny was evaluated using the Hasegawa-Kishino-Yano model (Hasegawa et al., 1985). Support for the nodes derived in these reconstructions was evaluated by bootstrapping using 1,000 replicates (Felsenstein, 1985).

The congruence among the different phylogenetic reconstructions was evaluated using Shimodaira–Hasegawa (SH) (Shimodaira and Hasegawa, 1999) and expected-likelihood weight (ELW) tests as implemented in TreePuzzle version 5.2 (Schmidt et al., 2002; Strimmer and Rambaut, 2002).

## Results

### Core genome

We obtained almost complete genome sequences of 38 *V. vulnificus* strains (Table 1). The CGS was inferred from these genomes and additional genomes taken from the database (80 in total). The analysis included strains of the three Bts, isolated from a variety of hosts and habitats all over the world (Table 1). The main characteristics of the analyzed genomes and the CGS as well as the number of SNPs identified per chromosome are detailed in Tables 2, 3, respectively. The average gene identities in the CGS were 91 and 89% for chromosome I and II, respectively, which supports previous observations pertaining to the greater variability of chromosome II in *Vibrio* spp. (Kirkup et al., 2010).

**Table 2:** Some general data of the genome and core genome of the species (CGS) *V. vulnificus*.

	**Average Genome size in bp (number of genes)**	**Core**			
		**Size (bp)**	**Genes**	**SNP**	**%G+C**
Chromosome I	3,286,174 (3,103)	1,396,961	1,304	132,027	46.9
Chromosome II	1,803,986 (1,571)	519,724	414	45,027	46.8
Chromosome I+II	5,090,160 (4,674)	1,916,685	1,718	177,054	46.9

**Table 3:** Number of SNPs sites per lineages identified in the core genome of the species (CGS).

**Lineage**	**Chromosome I**			**Chromosome II**		
	**Specific SNPs**	**% Specific SNPs of total SNPs**	**% Specific SNPs of Core Genome**	**Specific SNPs**	**% Specific SNPs of total SNPs**	**% Specific SNPs of Core Genome**
L1	83,107	62.95	5.95	28,193	62.61	5.42
L2	63,308	47.95	4.53	21,870	48.57	4.21
L3	25,034	18.96	1.79	9,619	21.36	1.85
L4	15,989	12.11	1.14	6,090	13.53	1.17
L5	132,027	100	9.45	45,027	100	8.66
L1-L2	117,305	88.85	8.4	39,924	88.67	7.68
L1-L3	91,163	69.05	6.53	30,933	68.7	5.95
L1-L4	89,724	67.96	6.42	30,230	67.14	5.82
L1-L5	87,874	66.56	6.29	29,778	66.13	5.73
L2-L3	74,594	56.5	5.34	26,214	58.22	5.04
L2-L4	85,808	64.99	6.14	29,097	64.62	5.6
L2-L5	71,527	54.18	5.12	24,968	55.45	4.8
L3-L4	52,708	39.92	3.77	18,246	40.52	3.51
L3-L5	34,277	25.96	2.45	12,514	27.79	2.41
L4-L5	42,081	31.87	3.01	13,676	30.37	2.63

The genes of the CGS and the associated metabolic pathways are shown in Tables S2, S3, respectively. The genes present in all the strains that did not match the criteria used to define the CGS (spanning less than 80% of the length and showing less than 70% identity with respect to the homologous gene in the reference YJ016 genome) are shown in Table S4. The CGS includes practically all of the genes for glycolysis, TCA cycle and pentose phosphate pathway, aerobic and anaerobic respiration, nitrate respiration, as well as for biosynthesis of metabolic intermediates, cofactors, nucleotides, amino acids and cell building blocks (Tables S2, S3). The CGS also includes genes involved in survival in different environments (water, animal intestine, chitinous surfaces), such as genes for resistance to different stressors (e.g., cold, heat, toxic oxygen forms, antimicrobials, and tellurite), genes for chitin degradation (i.e., various chitinases) and genes for surface colonization [e.g., pilus MSHA (mannose sensitive hemagglutination) and flagellum biogenesis]. It is generally assumed that genes on the *Vibrio* chromosome II have specific environmental functions related to habitat adaptation (Xu et al., 2003). Regarding the metabolic pathways, all of the common genes for general metabolic pathways such as butanoate metabolism, glycerolipid metabolism, peptidoglycan biosynthesis and pyrimidine metabolism are located on chromosome I while the genes for biosynthesis of siderophores, which could be related to habitat adaptation, are in the chromosome II but together with other metabolic genes not clearly related to habitat adaptation (i.e., fatty acid biosynthesis/elongation, glycerophospolipid metabolism, glyoxylate/dicarboxylate metabolism, nicotinate/nicotinamide metabolism, porphyrin metabolism, and valine/leucine/isoleucine metabolism) (Table S3). Regarding to survival genes, genes for the flagellum and MSHA pilus are located in chromosome I while those for the Flp/Tad pilus are located in chromosomes I and II (Table S2). In conclusion, there is no clear relationship between chromosome localization and habitat adaptation for the CGS-genes.

**Figure 1:**
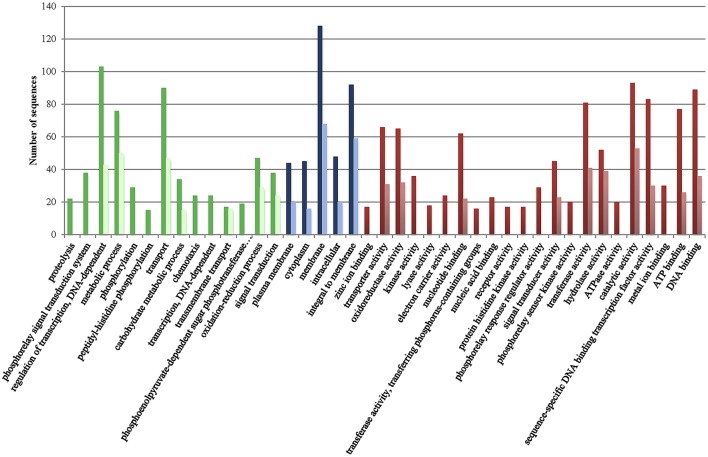
Gene ontology terms distribution of the core genome of the species (CGS) in chromosomes I (dark color) and II (light color). Green, biological process (max. level 15); Blue, cellular component (max. level 15); Red, Molecular Function (max. level 15).

Figure 1 presents the distribution of gene ontology terms. Most of the genes in the CGS encode proteins associated with cell membranes that are related with regulation of transcription, transport or metabolic/oxido-reduction process and present catalytic/hydrolase, transferase or transcription-factor activity (Figure 1). Again, many functions associated to CGS genes in chromosome I were not found in chromosome II such as kinase activity, metal ion transport and phospho-relay response regulator activity, etc. (Figure 1). In addition, no CGS genes related to proteolysis, phospho-relay signal transduction, carbohydrate transport, phosphorylation, ATP catabolic process, chemotaxis, biosynthesis, DNA recombination and repair, and phosphoenolpyruvate-dependent sugar phosphotransferase system were identified in chromosome II (Figure 1).

To determine the CGP, the plasmid was reconstructed from the sequenced genomes of the Bt2 strains indicated in Table 1. According to the phylogenetic tree (**Figure 3**), we found six variants of the plasmid, two previously described (types II and IV) (Lee et al., 2008; Roig and Amaro, 2009) and four new ones (Table 4). The list of genes in the CGP is shown in Table S5. The CGP includes three virulence genes, two host-specific, *vep07* and *ftbp* (fish transferrin binding protein), involved in resistance to and growth in eel serum, respectively (unpublished results, Pajuelo et al., 2015), and one host-nonspecific, *rtxA1*. This is a mosaic gene related with resistance to phagocytosis by murine and eel phagocytes (Lee et al., 2013a; Satchell, 2015) that presents at least seven different forms in *V. vulnificus* (Roig et al., 2011; Satchell, 2015).

**Table 4:** Virulence plasmid types and main characteristics.

**pVvbt2 type**	Strains^*^	**% identity whole sequence**	**% identity core**	**Max size**	**Min size**	**Core size**	**GC**
I	95-87, 95-86, A14	98.6	100	65,910	65,375	46,446	43.4
II	R02, CECT4999, CECT5763	80.2	100	68,446	66,603	46,446	43.3
III	94-8-112, 96-0426-1-4C	81.5	99.7	64,359	63,933	46,446	43.8
IV	CECT898, CECT4865, PD-2-51, Rae3, CECT4604, CECT4866, CIP8190	88.1	100.0	68,448	66,946	46,446	43.5
V	CECT7030, CECT5769	90.2	97.9	66,249	60,284	46,446	43.4
VI	95-8-161	100	100	63,933	63,933	46,446	43.3

* *Serovar E strains: R02, CECT4999, CECT5763, 94-8-112, CECT898, CECT4865, PD-2-51, Rae3, CECT4604, CECT4866, CIP8190. Serovar A strains: CECT7030, CECT5769, A14. Serovar I, 95-87, 95-86, 95-8-161. Non typable, 96-0426-1-4C*.

### Phylogenomic analysis

To root the *V. vulnificus* phylogenetic tree within the genus, we first reconstructed a phylogenetic tree from the closed genomes of 15 strains belonging to seven *Vibrio* species (*V. parahaemolyticus, V. anguillarum, V. algynolyticus, V. campbellii, V. furnissii, V. cholerae*, and *V. splendidus-clade*) (main genome characteristics shown in Table S1) together with 27 selected *V. vulnificus* genomes (Table 1). Maximum-likelihood (ML) trees for chromosome I, II and I+II were reconstructed based on the common genes of these *Vibrio* spp. (Figure S1). The ML trees showed *V. vulnificus* as a very compact and independent group. Next, we inferred the phylogeny of the species from the 80 *V. vulnificus* genomes indicated in Table 1 by using ML reconstruction obtained from the SNPs of coding regions.

**Figure 2:**
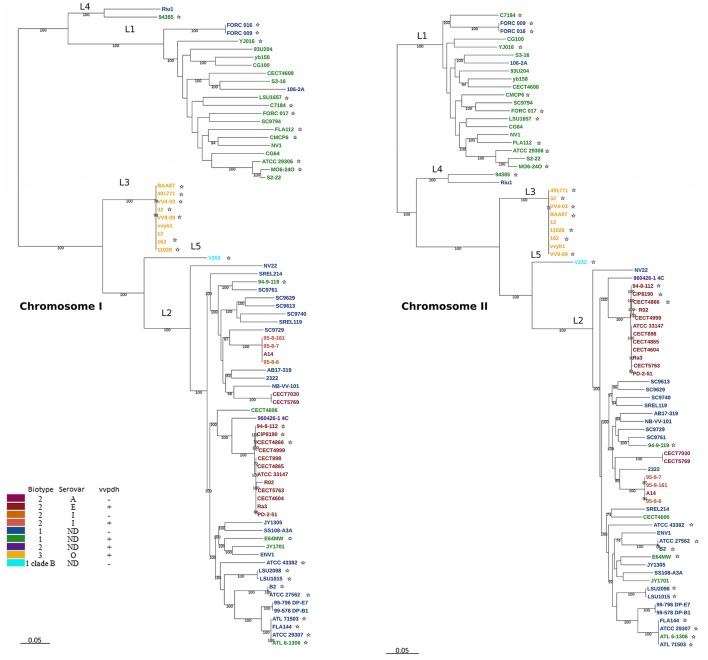
*V. vulnificus* phylogeny reconstructed from single nucleotide polymorphisms (SNPs) of the coding regions in the CGS. *V. vulnificus* phylogeny based on single nucleotide polymorphisms (SNPs) of the coding regions in the core genome of the species (CGS). Maximum-likelihood tree derived using the generalized time-reversible model (GTR+G+I) model of evolution. Bootstrap support values higher than 70% are indicated in the corresponding nodes. ^*^Human clinical isolate.

The phylogenetic trees for *V. vulnificus* are shown in Figure 2 and Figure S2. The rooted *V. vulnificus* phylogenetic reconstructions for chromosomes I and II were compared by congruence tests (Table 5). The results clearly indicate that chromosomes I and II were not congruent with one another, which strongly suggests that both chromosomes have suffered inter and/or intra chromosomal rearrangement since the emergence of the *V. vulnificus* ancestor. In fact, several strains changed their relationships in the tree for each chromosome (Figure 2). Among them, we highlight the well-known human clinical isolates C7184, YJ016, and CMCP6 (Figure 2). Despite the global incongruence between the two chromosomal phylogenies, all the phylogenetic trees divided the species into five well-defined, highly supported lineages, two of them (L4 and 5) including only a few strains. No strain changed lineage between the two trees (Figure 2).

**Table 5:** Summary of Shimodaira-Hasegawa (SH) and Expected Likelihood Weight (ELW) tests.

**Alignment**	**Topology**	**log-L**	**p-1sKH**		**p-SH**		**c-ELW**		**p-AU**	
ChrI	ChrI	−2157881.655	1.000	+	1.000	+	1.000	+	1.000	+
	ChrII	−2179900.127	0.000	–	0.000	–	0.000	–	0.000	–
ChrII	ChrI	−761721.329	0.000	–	0.000	–	0.000	–	0.000	–
	ChrII	−757275.779	1.000	+	1.000	+	1.000	+	1.000	+
Virulence Plasmid	ChrI	−73205.493	0	–	0	–	0	–	0.0019	–
	ChrII	−73221.068	0	–	0	–	0	–	0.0009	–
Virulence Plasmid	Virulence Plasmid	−72555.771	1.000	+	1.000	+	1.000	+	0.9977	+
ChrI	ChrI	−340807.458	0.942	+	1.000	+	0.955	+	0.972	+
	ChrII	−340840.343	0.058	+	0.496	+	0.0454	–	0.028	–
	Virulence Plasmid	−405217.661	0	–	0	–	0	–	0.005	–
ChrII	ChrI	−149737.006	0.043	–	0.472	+	0.014	–	0.078	–
	ChrII	−149703.356	0.957	+	1.000	+	0.986	+	0.998	+
	Virulence Plasmid	−178030.177	0	–	0	–	0	–	0.0066	–

*The columns show the results and p-values of the following tests: 1sKH—one sided KH test based on pairwise SH tests (Kishino and Hasegawa, 1989; Shimodaira and Hasegawa, 1999; Goldman et al., 2000); SH, Shimodaira–Hasegawa test (Shimodaira and Hasegawa, 1999); ELW, Expected Likelihood Weight (Strimmer and Rambaut, 2002); p-AU—approximately unbiased test (Shimodaira, 2002). p < 0.05. +, positive congruence; –, negative congruence*.

The pairwise identity for the strains of all the lineages was 82.5% (Table 6). Lineage 1 (L1) (pairwise identity 93.1%) includes clinical (50% of isolates) and environmental (50% of isolates) Bt1 isolates from the USA, South Korea, Taiwan, Israel, and Spain. The clinical L1 isolates were recovered from human infections in the USA, South Korea, and Taiwan, with 80% of these derived from blood (related to primary septicemia, when the etiology is known), 10% from stools (related to gastroenteritis) and 10% from wound samples. The environmental L1 isolates were recovered from oysters (40%), water (30%), and fish (30%) in Spain, Israel, Taiwan, South Korea, and the USA. Interestingly, the strain yv158, although environmental, belongs to a previously described highly clonal group (clade A), which includes both environmental and clinical isolates (wound samples), all of *vcg* C-type (clinical type according to the polymorphism in virulence correlated gene; Rosche et al., 2005), supporting the potential virulence of this specific strain (Broza et al., 2012).

**Table 6:** Global identity of groups and lineages.

**Name**	**% Pairwise Identity**
Whole	82.5
Biotype 3	97.8
Biotype 2	94.8
Lineage 1	93.1
Lineage 2	87.4
Lineage 3	97.8
Lineage 4	98.7
Lineage 5	100
Biotype 2 SerA	91.0
Biotype 2 SerE	97.7
Biotype 2 SerI	100.0

L2 (pairwise identity 87.4%) includes Bt1 and Bt2 strains from environmental sources (39.6% of the isolates; 21% from water, 21% from fish, 53.5% from seafood and 4.5% from sediment), diseased humans [29.2% of the isolates; 57% from human blood (none of them with known etiology) and 43% from wounds] and diseased fish (31.2%) origins. All of the zoonotic strains (Bt2-SerE), regardless of their source (environment, diseased animal or diseased human), country (France, Australia, Denmark and Spain) or year of isolation (from 1980 to 2004), cluster in a highly homogeneous group (pairwise identity 97.7%). The non-typeable Bt2 strain (960426-1 4C) formed a subgroup together with the SerE clonal-complex while SerI and A strains formed another subgroup both within L2 (Figure 2).

L3 includes all the Bt3 strains, which form a highly homogeneous group (pairwise identity 97.8%). These Bt3 strains were isolated in Israel from outbreaks of human vibriosis associated with the handling of farmed-tilapia (year of isolation, 1996–2003) and from aquaculture fishponds of tilapia (2002–2004). In consequence, all of them are associated to the aquaculture industry.

Interestingly, all human clinical cases of known etiology caused by L2 and L3 isolates in Europe and Israel were related to farmed-fish handling regardless the Bt of the isolate.

L4 (similarity 98.7%) is formed by two Spanish isolates from the Ebro-Delta (a nature park by the Mediterranean Sea) area, one from seawater and the other from a human clinical (leg wound) case, both of Bt1. Finally, L5 is formed by a unique isolate of Bt1 from Israel and clinical origin that is considered as representative of a highly virulent clone designated as Clade B (Raz et al., 2014; Efimov et al., 2015).

**Figure 3:**
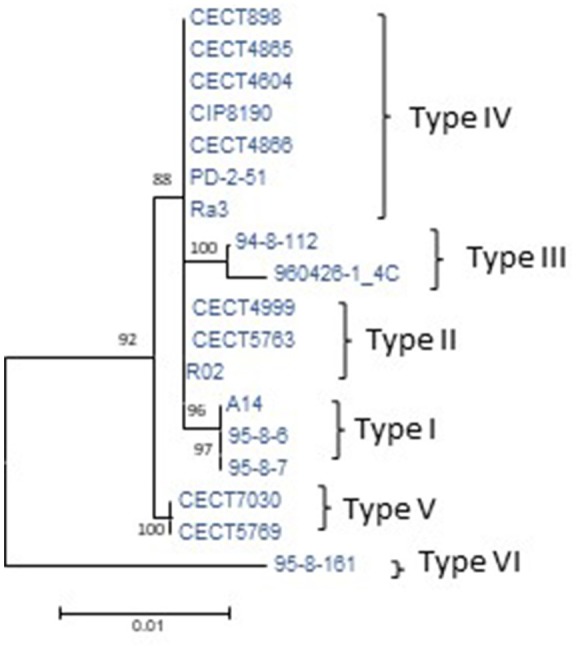
Phylogeny of virulence plasmid pVvbt2 based on SNPs in the core genome of the plasmid (CGP). Maximum-likelihood tree using the Hasegawa-Kishino-Yano model of evolution with gamma distribution and invariant sites. Bootstrap support values higher than 70% are indicated in the corresponding nodes. The different types of plasmids detected and described in Table 4 are marked to the right of the figure.

The phylogenetic tree for pVvBt2 based on the CGP shows relationships among isolates that did not match the previous phylogenetic relationships (Figures 2, 3). Further, the congruence analyses revealed that phylogenetic trees from the CGP and CGS were not congruent to each other, suggesting a different evolutionary history for the plasmid and the chromosomes (Table 5).

### Virulence-related genes and VCGS

We searched for human virulence-related genes described in the literature for *V. vulnificus*, and we found that 75% of them were present in the CGS. This finding allowed us to define the human virulence-related core genome (VCGS) (Table S6). The VCGS includes genes for the flagellum, capsule, LPS- and cell-wall biosynthesis, motility, hemolysin, and proteases (such as VvhA and a collagenase), resistance to human serum (*trkA;* Chen et al., 2004), heme uptake, biosynthesis and uptake of vulnibactin, several genes for transcriptional regulators such as Fur, and finally genes for MARTX (Multifunctional Autoprocessive Repeat in Toxin) transport and modification systems, genes that are duplicated in pVvbt2 (Chakrabarti et al., 1999; Horstman et al., 2004; Yu-Chung et al., 2004; Gulig et al., 2005; Bogard and Oliver, 2007; Alice et al., 2008; Lee et al., 2008; Oh et al., 2008; Brown and Gulig, 2009; Liu et al., 2009; Chen and Chung, 2011; Kim et al., 2011; Oliver, 2015; Satchell, 2015). Remarkably, genes for several collagenases and chitinases were found in both chromosomes while the MARTX operon and *vvhA* were located on chromosome II.

## Discussion

The core genome has been demonstrated to be an optimum data set for determining the phylogeny of a bacterial species since it primarily includes the essential genes of a species and excludes the non-essential ones, such as those present in MGE (mobile genetic element: genomic islands, prophages and plasmids) (Juhas et al., 2009; Segerman, 2012; Wolf et al., 2013). In the current study, we have used this approach to analyze the phylogeny of *V. vulnificus* and compare the phylogenetic groups with the current Bts of the species.

Our phylogenomic analysis suggests that *V. vulnificus* has diverged in five well-defined and separate lineages that do not correspond with the current Bts. L1 is formed by the most dangerous strains from a public health perspective. All of them correspond to Bt1 and were mostly isolated from human blood in North America and Asia, presumably from primary septicemia cases after ingestion of raw seafood. L2 and L3 comprise strains of the three Bts, mostly isolated from fish-farming related environments, including humans infected through handling of farm-fish in Europe and Israel (Bisharat et al., 1999; Haenen et al., 2013).

L2 includes Bt1 and Bt2 strains. Sanjuán et al. (2011) proposed that Bt2 is a polyphyletic group subdivided in Ser-related subgroups, one of which is a clonal complex (Bt2-SerE). Our phylogenomic study confirms that Bt2 is polyphyletic and that the SerE-subgroup is highly homogeneous (identity value of 97.7%). Bt2 was defined in 1982 based on the differential properties of the first fish-pathogenic strains, all of which belonged to SerE and were isolated from eel-farms in Japan (Muroga et al., 1976a,b; Tison et al., 1982). Later, Bt2-SerE isolates were recovered from human infections registered in the USA and Europe, some of them related to zoonotic cases and others of unknown etiology, as well as from different epizootic events of high mortality affecting different farmed-eels in Europe. Bt2-SerA and SerI emerged simultaneously in Spanish and Danish farms after the industry initiated the change from brackish- to fresh-waters in order to control the severity of vibriosis outbreaks due to Bt2-SerE as well as the probability of human infections (Fouz and Amaro, 2003). These new serovars are adapted to infect through and to survive in fresh-waters (Fouz et al., 2006).

All of the analyzed Bt2 strains contained the virulence plasmid pVvbt2, SerE strains present three variants of the plasmid, the two variants previously described (Lee et al., 2008) and a new one (Table 4). SerA and I strains showed three new variants (Table 4). It was previously hypothesized that Bt2 emerged in fish farms after acquisition of pVvbt2 by different clones of Bt1 strains (Sanjuán et al., 2011). To test this hypothesis, we compared the chromosomal phylogenetic trees reconstructed for Bt2 strains from the CGS with those from the CGP and found that they were not congruent. This result strongly supports the hypothesis of Sanjuán et al. (2011) and suggests that pVvbt2 has been acquired independently by different clones within L2. One of these plasmid-carrier clones successfully amplified in eel-farms and spread to other places and countries, probably in carrier fishes, giving rise to the worldwide expanded, current clonal complex. This clonal complex is supposed to be zoonotic because there are clinical Bt2-SerE isolates related to diseased fish handling and because all the fish and environmental isolates examined to date are virulent for both fish and mice (Sanjuán and Amaro, 2004).

L3 includes all Bt3 strains regardless of their origin (human infections related to fish-farms or environmental), which constitute a clonal group. Bt3 emerged in Israel in 1990 in farms of tilapia and is the only one that has produced outbreaks of human infections, all of them through severe wound infections or secondary septicemia cases (Bisharat et al., 1999). By using different genomic approaches, Raz et al. (2014) and Koton et al. (2014) have hypothesized that Bt3 emerged in the nutrient-enriched environment represented by the aquaculture industry from a Bt1 ancestor that acquired a rather small number of genes from different donors, leading to a change in biotype. The proposed ancestor was v252, a representative strain from a highly virulent clade designated as clade B that shares high similarity and appeared close to Bt3 (Raz et al., 2014; Efimov et al., 2015). Our analysis does not support this hypothesis. Instead, clade B shares the closest common ancestor with L2 and not with L3 in spite of having been isolated from the same “melting pot” where biotype 3 was evolved, i.e., aquaculture fish farms in Israel.

Comparisons of the core genome between clinical and environmental strains of the closely related species *V. cholerae* reveal that this species is divided into two linages, with most of the epidemic strains appearing closely related, regardless of their geographical origins (Eppinger et al., 2014). The only clonal *V. vulnificus* group with a worldwide distribution is that formed by Bt2-SerE strains, a group that combines the ability to infect fish with that of infecting humans and of surviving in the environment without nutrients for years in a viable but non-culturable state (Marco-Noales et al., 1999). Moreover, in *V. cholerae* the ability to cause cholera epidemics lies on mobile genetic elements, such as phages and pathogenicity islands, that carry the genes encoding the cholera toxin, TCP pilus, etc. (Ramamurthy and Bhattacharya, 2011; Das et al., 2016). In contrast, our phylogenomic analysis, as well as those based on MLSA (Cohen et al., 2007; Sanjuán et al., 2011) and microarray hybridization (Raz et al., 2014), show that environmental and clinical strains of *V. vulnificus* are distributed throughout the phylogenetic lineages, regardless the Bt, country of origin, or year of isolation. This result is compatible with the hypothesis that essentially all *V. vulnificus* isolates, unlike *V. cholerae*, have the ability to infect humans. To confirm this, we investigated which virulence-related genes were present in the CGS and found that most of them belong to the core genome.

Summarizing, all of the phylogenetic reconstructions from the core genome of the species, the fish-virulence plasmid and the human-virulence genes strongly suggest that *V. vulnificus* emerged from an ancestor potentially virulent for humans that diverged in five lineages that do not correspond with the current Bts. Our results also highlight the importance of the aquaculture industry in the recent evolution and epidemic spread of the species and, finally, support the intra-specific classification in lineages instead of in Bts as well as the inclusion of a pathovar grouping all fish pathogenic isolates for which we propose the name “piscis.”

## Author contributions

CA, FG-C, EF, and FR designed the work, FR, ES, and FG-C performed the phylogenomic analysis, YD-P, BF, CG, CB-A, PG, and SM discussed the preliminary results. CA and FR wrote the paper. All the authors contributed to the discussion and improvement of the MS.

### Conflict of interest statement

The authors declare that the research was conducted in the absence of any commercial or financial relationships that could be construed as a potential conflict of interest.

## Supplementary material

Figure S1 *Vibrio* phylogeny based on the concatenated whole core genome of seven *Vibrio* species with closed genomes together with selected *V. vulnificus* strains for chromosome I, chromosome II and chromosome I+II. Maximum-likelihood tree derived from the aligned regions by using the GTR+G+I model of evolution. Bootstrap support values higher than 70% are indicated in the corresponding nodes. Color code: green, Bt1 vvpdh+; blue; Bt1 vvpdh−; yellow; Bt3 vvpdh+; red; Bt2 vvpdh+; magenta; Br2 vvpdh−.

Figure S2 *V. vulnificus* phylogeny reconstructed from single nucleotide polymorphisms (SNPs) of the coding regions in the CGS for both chromosmes (ChrI+ChrII). *V. vulnificus* phylogeny based on single nucleotide polymorphisms (SNPs) of the coding regions in the core genome of the species (CGS). Maximum-likelihood tree derived using the generalized time-reversible model (GTR+G+I) model of evolution. Bootstrap support values higher than 70% are indicated in the corresponding nodes. ^*^Human clinical isolate.

Table S1 Characteristics of the Vibrio genomes used to define the core genome of the genus (CGG) according to the NCBI Databases.

Table S2 *V. vulnificus* core genome.

Table S3 Metabolic pathways associated to *V. vulnificus* core genome.

Table S4 *V. vulnificus* common genes that were not considered to be part of the Core Genome because its identity and length with respect to the reference sequence were lower than 70 and 80%, respectively.

Table S5 Core genes in the *V. vulnificus* plasmid pVvbt2 (CGP).

Table S6 *V. vulnificus* virulence genes in the core genome.
