# Corrigendum: Phylogeny of *Vibrio vulnificus* From the Analysis of the Core-Genome: Implications for Intra-Species Taxonomy

**DOI:** 10.3389/fmicb.2019.01904

**Published:** 2019-08-21

**Authors:** Francisco J. Roig, Fernando González-Candelas, Eva Sanjuán, Belén Fouz, Edward J. Feil, Carlos Llorens, Craig Baker-Austin, James D. Oliver, Yael Danin-Poleg, Cynthia J. Gibas, Yechezkel Kashi, Paul A. Gulig, Shatavia S. Morrison, Carmen Amaro

**Affiliations:** ^1^Estructura de Investigación Interdisciplinar en Biotecnología y Biomedicina BIOTECMED, University of Valencia, Valencia, Spain; ^2^Departmento de Microbiología y Ecología, Universidad de Valencia, Valencia, Spain; ^3^Biotechvana, Parc Cientific, Universitat de Valencia, Valencia, Spain; ^4^Joint Research Unit on Infection and Public Health FISABIO-Salud Pública and Universitat de Valencia-I2SysBio, Valencia, Spain; ^5^CIBEResp, National Network Center for Research on Epidemiology and Public Health, Instituto de Salud Carlos III, Valencia, Spain; ^6^Department of Biology and Biochemistry, University of Bath, Bath, United Kingdom; ^7^Centre for Environment, Fisheries and Aquaculture Science, Weymouth, United Kingdom; ^8^Department of Biological Sciences, University of North Carolina at Charlotte, Charlotte, NC, United States; ^9^Duke University Marine Lab, Beaufort, NC, United States; ^10^Faculty of Biotechnology and Food Engineering, Technion–Israel Institute of Technology, Haifa, Israel; ^11^Department of Bioinformatics and Genomics, the University of North Carolina at Charlotte, Charlotte, NC, United States; ^12^Department of Molecular Genetics and Microbiology, University of Florida, Gainesville, FL, United States

**Keywords:** microbial evolution, pathogens, SNP, *Vibrio vulnificus*, core genome, virulence plasmid, pathovar, biotype

There is an error in the Funding statement. The first funding number is incorrect and should be AICO/2018/123. The authors apologize for this error and state that this does not change the scientific conclusions of the article in any way. The original article has been updated.

