# Supplementary figures and images for: Phylogeny of Vibrio vulnificus from the Analysis of the Core-Genome: Implications for Intra-Species Taxonomy
Source: Front Microbiol. 2018 Jan 5;8:2613. doi: 10.3389/fmicb.2017.02613 (PMC5765525; doi:10.3389/fmicb.2017.02613)

# Chromosome I

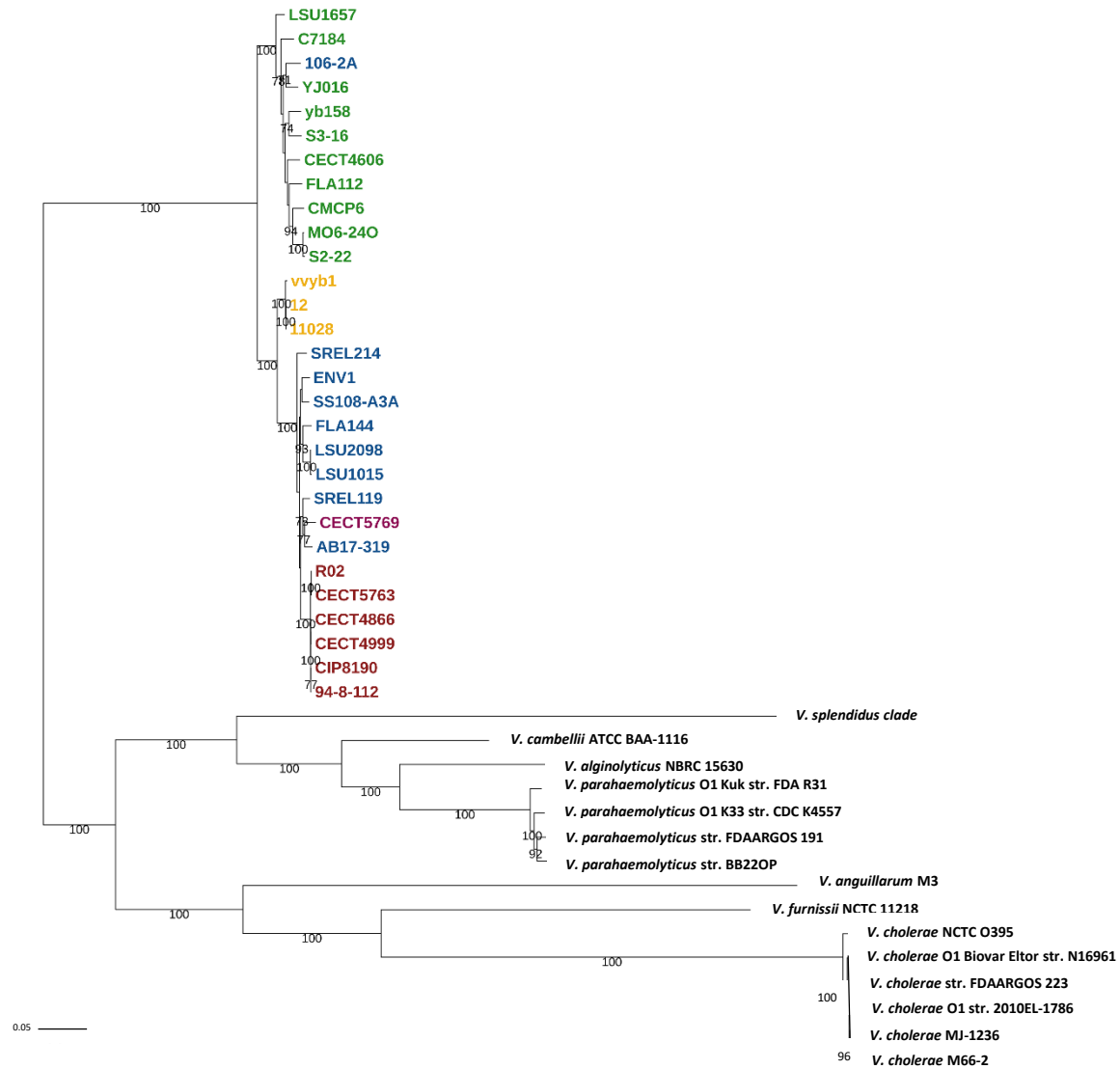

# Chromosome II

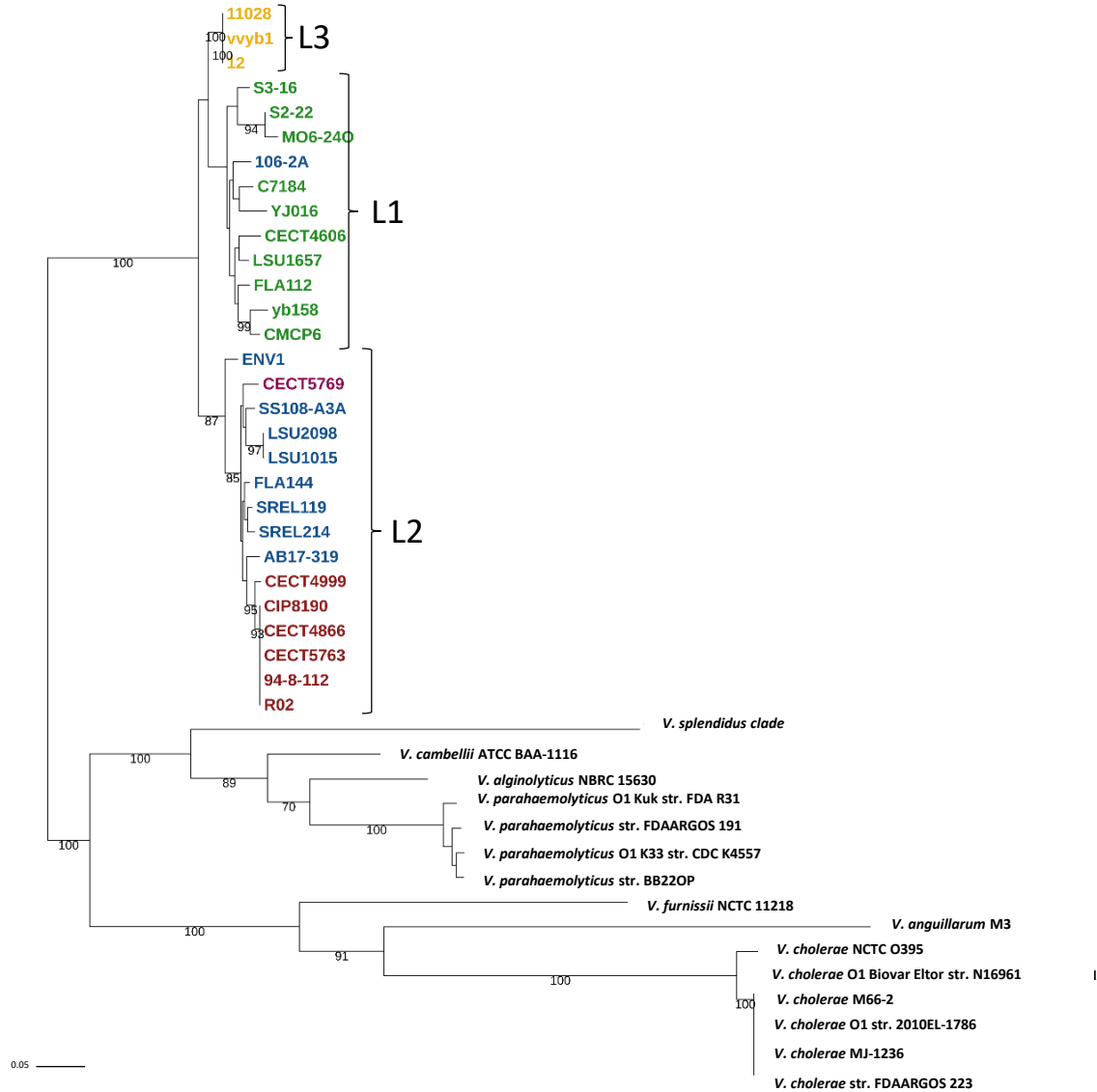

# Chromosome I+II

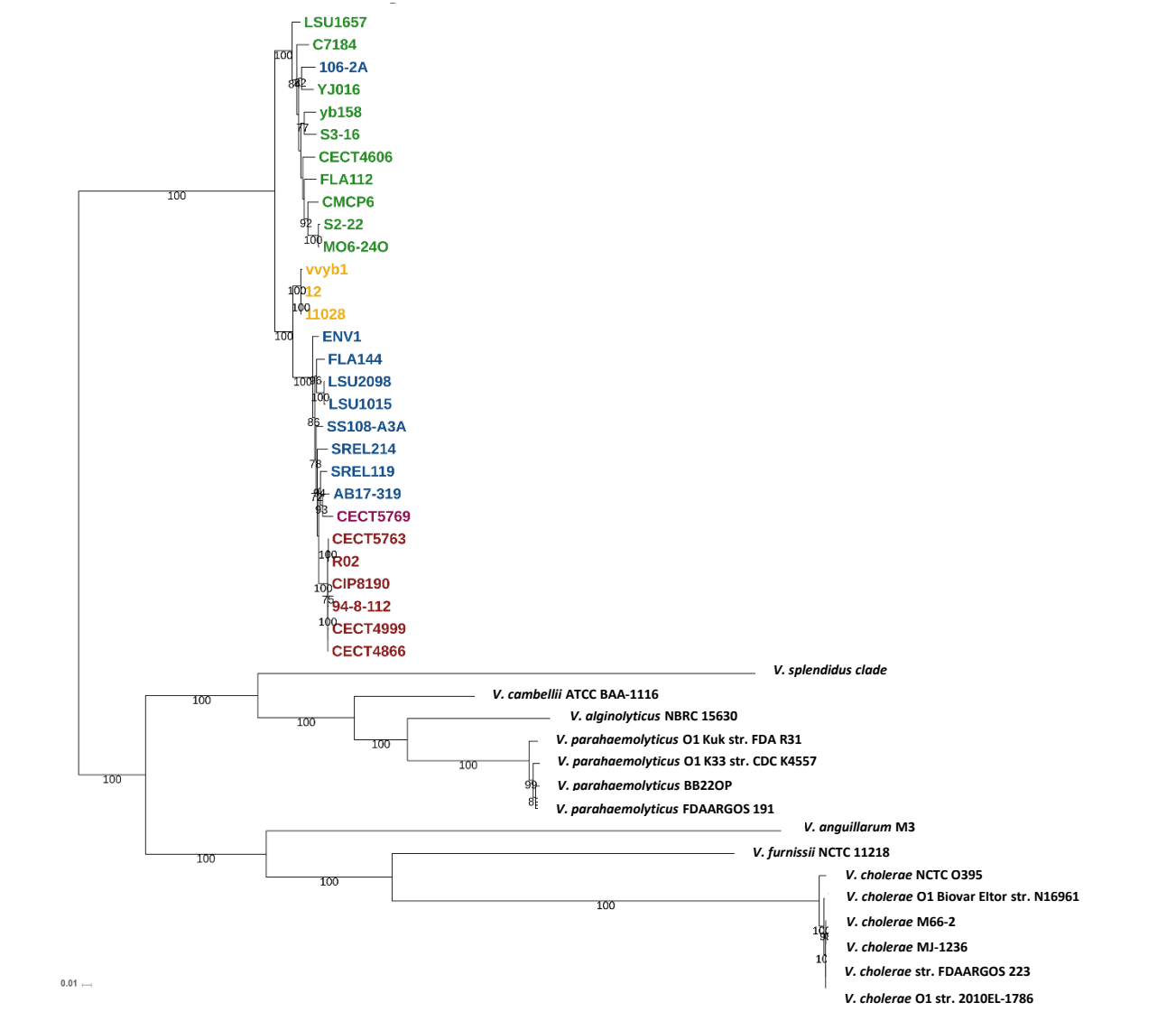

Supplement: Figure S1 — Vibrio phylogeny based on the concatenated whole core genome of seven Vibrio species with closed genomes together with selected V. vulnificus strains for chromosome I, chromosome II and chromosome I+II. Maximum-likelihood tree derived from the aligned regions by using the GTR+G+I model of evolution. Bootstrap support values higher than 70% are indicated in the corresponding nodes. Color code: green, Bt1 vvpdh+; blue; Bt1 vvpdh−; yellow; Bt3 vvpdh+; red; Bt2 vvpdh+; magenta; Br2 vvpdh−. [file Image1.PDF]

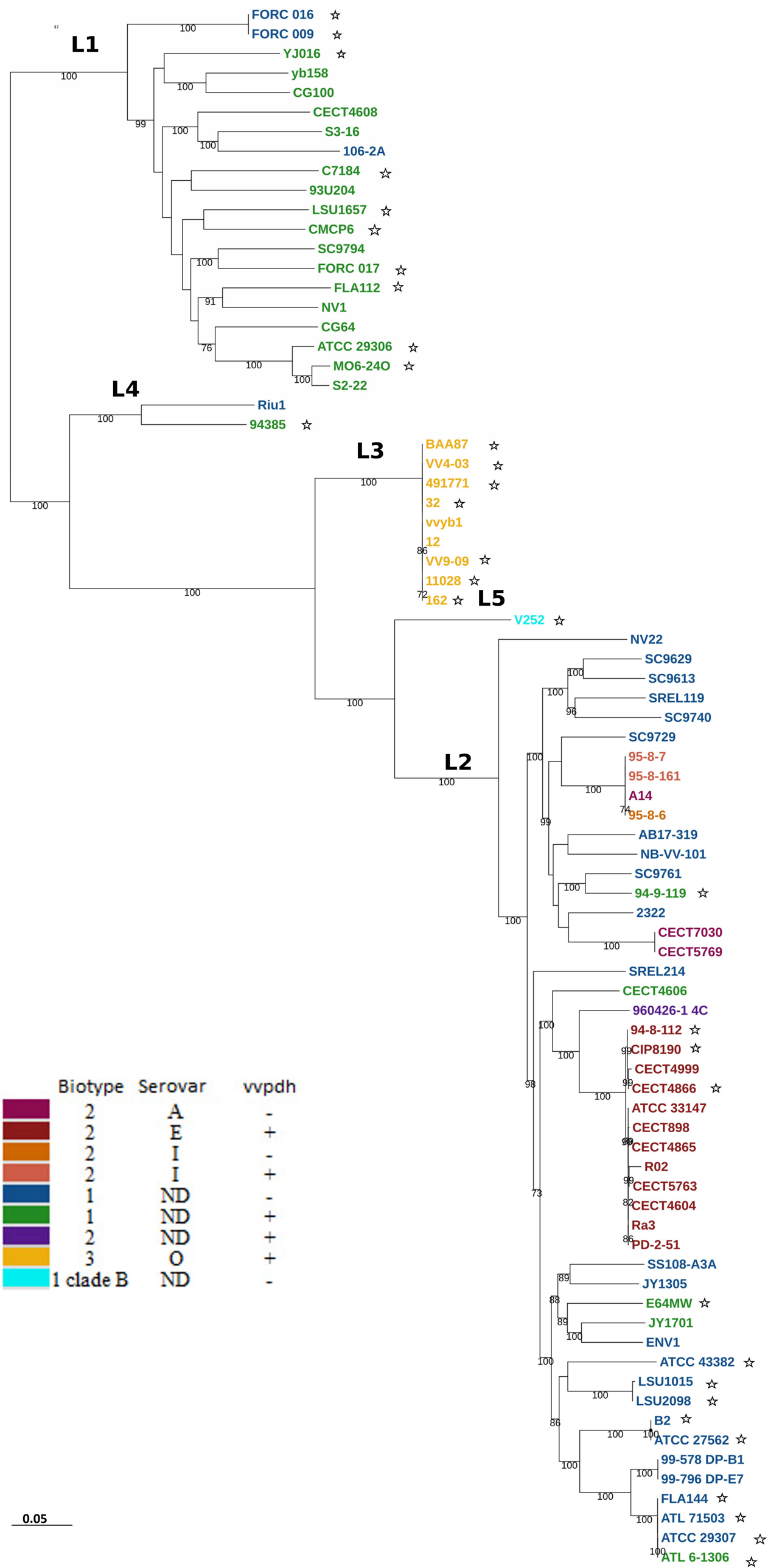

Supplement: Figure S2 — V. vulnificus phylogeny reconstructed from single nucleotide polymorphisms (SNPs) of the coding regions in the CGS for both chromosmes (ChrI+ChrII). V. vulnificus phylogeny based on single nucleotide polymorphisms (SNPs) of the coding regions in the core genome of the species (CGS). Maximum-likelihood tree derived using the generalized time-reversible model (GTR+G+I) model of evolution. Bootstrap support values higher than 70% are indicated in the corresponding nodes. *Human clinical isolate. [file Image2.PDF]
