# Supplementary material for: Phylogeny of Vibrio vulnificus from the Analysis of the Core-Genome: Implications for Intra-Species Taxonomy
Source: Front Microbiol. 2018 Jan 5;8:2613. doi: 10.3389/fmicb.2017.02613 (PMC5765525; doi:10.3389/fmicb.2017.02613)
Supplement: Table S1 — Characteristics of the Vibrio genomes used to define the core genome of the genus (CGG) according to the NCBI Databases. [file Table1.DOCX]

| Table S1: Characteristics of the *Vibrio* genomes used to define the core genome of the genus (CGG) according to the NCBI Databases | | | | | |  |
| --- | --- | --- | --- | --- | --- | --- |
| Species  serovar/biotype | Strain | Gene number  ChrI+ChrII | GC%  ChrI/ChrII | Chromosomes | Protein  ChrI + II | |
| *V. parahaemolyticus* | BB22OP | 3,210 + 1,678 | 45.4/45.2 | chr.I:NC_019955.1/CP003972.1 | 3,006 + 1,636 | |
|  |  |  |  | chr.II:NC_019971.1/CP003973.1 |  |  |
| *V. parahaemolyticus* | FDAARGOS 191 | 3,215 + 1,749 | 45.4/45.4 | chr.I:NZ_CP020427.1/CP020427.1 | 2,946 + 1,678 | |
|  |  |  |  | chr.II:NZ_CP020428.1/CP020428.1 |  |  |
| *V. parahaemolyticus* O1:K33 | CDC K4557 | 3,376 + 1,648 | 45.3/45.4 | chr.I:NC_021848.1/CP006008.1 | 3,053 + 1,612 | |
|  |  |  |  | chr.II:NC_021822.1/CP006007.1 |  |  |
| *V. parahaemolyticus* O1:Kuk | FDA R31 | 3,308 + 1,718 | 45.4/45.2 | chr.I:NC_021847.1/CP006004.1 | 3,074 + 1,642 | |
|  |  |  |  | chr.II:NC_021821.1/CP006005.1 |  |  |
| *V. alginolyticus* | NBRC 15630 | 3,141 + 1,612 | 44.7/44.7 | chr.I:NC_022349.1/CP006718.1 | 2,954 + 1,567 | |
|  |  |  |  | chr.II:NC_022359.1/CP006719.1 |  |  |
| *V. campbelli* | ATCC BAA-1116 | 3,595 + 2,105 | 45.5/45.3 | chr.I:NC_022269.1/CP006605.1 | 3,241 + 1,875 | |
|  |  |  |  | chr.II:NC_022270.1/CP006606.1 |  |  |
|  |  |  |  | plasmid:NC_022271.1/CP006607.1 |  |  |
| *V. tasmaniensis* | LGP32 | 3,007 + 1,349 | 44/43.6 | chr.I:NC_011753.2/FM954972.2 | 2,816 + 1,386 | |
|  |  |  |  | chr.II:NC_011744.2/FM954973.2 |  |  |
| *V. anguillarum* | M3 | 2,846 + 905 | 44.6/44.1 | chr.I:NC_022223.1/CP006699.1 | 2,626 + 839 | |
|  |  |  |  | chr.II:NC_022224.1/CP006700.1 |  |  |
|  |  |  |  | plasmid:NC_022225.1/CP006701.1 |  |  |
| *V. furnissii* | NCTC 11218 | 3,118 + 1,531 | 50.7/50.5 | chr.I:NC_016602.1/CP002377.1 | 2,921 + 1,491 | |
|  |  |  |  | chr.II:NC_016628.1/CP002378.1 |  |  |
| *V. cholerae* O395 | O 395 | 2,886 + 1,141 | 47.8/46.9 | chr.I:NC_009456.1/CP000626.1 | 2,730 + 1,088 | |
|  |  |  |  | chr.II:NC_009457.1/CP000627.1 |  |  |
| *V. cholerae* O1 biovar El Tor | N16961 | 2,690 + 1,003 | 47.7/46.9 | chr.I:NC_002505.1/AE003852.1 | 2,543 + 970 | |
|  |  |  |  | chr.II:NC_002506.1/AE003853.1 |  |  |
| *V. cholerae* | M66-2 | 2,693 + 1,061 | 47.8/47.0 | chr.I:NC_012578.1/CP001233.1 | 2,543 + 1,006 | |
|  |  |  |  | chr.II:NC_012580.1/CP001234.1 |  |  |
| *V. cholerae* | MJ-1236 | 2,955 + 1,108 | 47.5/46.7 | chr.I:NC_012668.1/CP001485.1 | 2,801 + 1,076 | |
|  |  |  |  | chr.II:NC_012667.1/CP001486.1 |  |  |
| *V. cholerae* | FDA-ARGOS 223 | 2,788 + 1,090 | 47.7/46.9 | chr.I:NZ_CP020408.1/CP020408.1 | 2,576 + 1,030 | |
|  |  |  |  | chr.II:NZ_CP020407.1/CP020407.1 |  |  |
| *V. cholerae* O1 | 2010EL-1786 | 2,822 + 1,050 | 47.7/47.0 | chr.I:NC_016445.1/CP003069.1 | 2.695 + 1,010 | |
|  |  |  |  | chr.II:NC_016446.1/CP003070.1 |  |  |
