# Supplementary material for: Phylogeny of Vibrio vulnificus from the Analysis of the Core-Genome: Implications for Intra-Species Taxonomy
Source: Front Microbiol. 2018 Jan 5;8:2613. doi: 10.3389/fmicb.2017.02613 (PMC5765525; doi:10.3389/fmicb.2017.02613)
Supplement: Table S5 — Core genes in the V. vulnificus plasmid pVvbt2 (CGP). [file Table5.DOCX]

| **Table S5**: Core genes in the *V. vulnificus* plasmid pVvbt2 (CGP) | | |
| --- | --- | --- |
| Gene | Description | % identity |
| *parA* | plasmid partitioning protein | 100.000 |
| *pilT* | putative PilT protein | 100.000 |
| *rtxA* | RTX toxin RtxA | 83.000 |
| *rtxB* | RTX toxin transporter | 100.000 |
| *rtxC* | RTX toxin acyltransferase family | 100.000 |
| *rtxD* | RTX toxin transporter | 100.000 |
| *rtxE* | ABC-type bacteriocin/lantibiotic exporters | 100.000 |
| *traI* | putative conjugative transfer protein TraI | 100.000 |
| *VVCP005* | transposase | 100.000 |
| *VVCP006* | transposase | 99.038 |
| *VVCP007* | RTX toxins and related Ca2+-binding protein | 100.000 |
| *VVCP008* | hypothetical protein | 100.000 |
| *VVCP009* | putative transposase | 100.000 |
| *VVCP011* | hypothetical protein | 100.000 |
| *VVCP012* | hypothetical protein | 100.000 |
| *VVCP013* | hypothetical protein | 100.000 |
| *VVCP014* | putative cytoplasmic protein | 100.000 |
| *VVCP015* | hypothetical protein | 100.000 |
| *VVCP019* | resolvase | 100.000 |
| *VVCP020* | Fish transferrin binding protein (Ftbp) | 100.000 |
| *VVCP021* | transposase and inactivated derivatives | 100.000 |
| *VVCP022* | putative transposase | 100.000 |
| *VVCP023* | transthyretin | 100.000 |
| *VVCP024* | hypothetical protein | 100.000 |
| *VVCP025* | hypothetical protein | 100.000 |
| *VVCP027* | putative transposase | 100.000 |
| *VVCP028* | hypothetical protein | 100.000 |
| *VVCP029* | hypothetical protein | 100.000 |
| *VVCP030* | hypothetical protein | 100.000 |
| *VVCP031* | DNA-binding protein RDGA | 99.841 |
| *VVCP035* | peptide chain release factor 1 | 100.000 |
| *VVCP038* | putative transposase | 100.000 |
| *VVCP040* | cysteinyl-tRNA synthetase | 100.000 |
| *VVCP042* | LtrC-like protein | 100.000 |
| *VVCP043* | hypothetical protein | 100.000 |
| *VVCP044* | transcriptional regulator | 100.000 |
| *VVCP045* | hypothetical protein | 100.000 |
| *VVCP046* | hypothetical protein | 100.000 |
| *VVCP047* | hypothetical protein | 100.000 |
| *VVCP048* | type I restriction-modification system methyltransferase subunit | 100.000 |
| *VVCP049* | hypothetical protein | 100.000 |
| *VVCP052* | truncated transposase | 99.029 |
| *VVCP053* | hypothetical protein | 99.200 |
