# Supplementary material for: Phylogeny of Vibrio vulnificus from the Analysis of the Core-Genome: Implications for Intra-Species Taxonomy
Source: Front Microbiol. 2018 Jan 5;8:2613. doi: 10.3389/fmicb.2017.02613 (PMC5765525; doi:10.3389/fmicb.2017.02613)
Supplement: Table S6 — V. vulnificus virulence genes in the core genome. [file Table6.DOCX]

| **Table S6**: *Vibrio vulnificus* virulence genes in the core genome | | | | |
| --- | --- | --- | --- | --- |
|  | **Name** | **Aliasses** | **Full Name/ Function** | |
| ChroI Core Virulence genes | *cca* | VV0571 | multifunctional tRNA nucleotidyl transferase/2'3'-cyclic phosphodiesterase/2'nucleotidase/phosphatase | ^a^ |
|  | *cpxP* | VV3112 | repressor CpxP | |
|  | *djlA* | VV0482 | Dna-J like membrane chaperone protein | ^a^ |
|  | *dnaE* | VV2541 | DNA polymerase III subunit alpha | |
|  | *dnaK* | VV0832 | molecular chaperone DnaK | ^a^ |
|  | *flgA* | VV0957 | flagellar basal body P-ring biosynthesis protein FlgA | ^a^ |
|  | *flgC* | VV0961 | flagellar basal body rod protein FlgC | ^a^ |
|  | *fliA* | VV2464 | flagellar biosynthesis sigma factor | |
|  | *fliP* | VV2471 | flagellar biosynthesis protein FliP | ^a^ |
|  | *fliQ* | VV2470 | flagellar biosynthesis protein FliQ | ^a^ |
|  | *fliR* | VV2469 | flagellar biosynthesis protein FliR | ^a^ |
|  | *glmU* | VV3249 | bifunctional N-acetylglucosamine-1-phosphate uridyltransferase/glucosamine-1-phosphate acetyltransferase | |
|  | *greA* | VV2718 | transcription elongation factor GreA | |
|  | *greB* | VV0235 | transcription elongation factor GreB | ^a^ |
|  | *holA* | VV0909 | DNA polymerase III subunit delta | ^a^ |
|  | *lspA* | VV0688 | lipoprotein signal peptidase | ^a^ |
|  | *murG* | VV0614 | UDPdiphospho-muramoylpentapeptide beta-N- acetylglucosaminyltransferase | |
|  | *nusA* | VV2709 | transcription elongation factor NusA | ^a^ |
|  | *rho* | VV3181 | transcription termination factor Rho | |
|  | *rpoB* | VV3159 | DNA-directed RNA polymerase subunit beta | ^a^ |
|  | *rpoZ* | VV0242 | DNA-directed RNA polymerase subunit omega | ^a^ |
|  | *rseB* | VV2836 | periplasmic negative regulator of sigmaE | ^a^ |
|  | *sspA* | VV0599 | stringent starvation protein A | ^a^ |
|  | *VV0012* | VV0012 | DNA polymerase III subunit beta | ^a^ |
|  | *VV0067* | VV0067 | Zn-dependent oligopeptidase | |
|  | *VV0069* | VV0069 | DNA-binding transcriptional regulator AsnC | ^a^ |
|  | *VV0115* | VV0115 | RNA polymerase factor sigma-32 | ^a^ |
|  | *VV0123* | VV0123 | LexA repressor | ^a^ |
|  | *VV0186* | VV0186 | DNA polymerase I | |
|  | *VV0245* | VV0245 | periplasmic protein TonB2 | ^a^ |
|  | *VV0246* | VV0246 | tonB system transport protein ExbD2 | ^a^ |
|  | *VV0247* | VV0247 | tonB system transport protein ExbB2 | ^a^ |
|  | *VV0399* | VV0399 | DNA-directed RNA polymerase subunit alpha | ^a^ |
|  | *VV0448* | VV0448 | RNA polymerase factor sigma-54 | ^a^ |
|  | *VV0452* | VV0452 | 3-deoxy-D-manno-octulosonate 8-phosphate phosphatase | ^a^ |
|  | *VV0561* | VV0561 | RNA polymerase sigma factor RpoD | |
|  | *VV0590* | VV0590 | protease DegS | ^a^ |
|  | *VV0655* | VV0655 | uracil-DNA glycosylase | |
|  | *VV0703* | VV0703 | membrane protein | |
|  | *VV0721* | VV0721 | collagenase | ^a^ |
|  | *VV0762* | VV0762 | aminopeptidase B | ^a^ |
|  | *VV0833* | VV0833 | chaperone protein DnaJ | |
|  | *VV0850* | VV0850 | deacylase | ^a^ |
|  | *VV0852* | VV0852 | aminoacyl-histidine dipeptidase | ^a^ |
|  | *VV1152* | VV1152 | protease IV |  |
|  | *VV1163* | VV1163 | ktrA protein | ^a^ |
|  | *VV1172* | VV1172 | V10 pilin |  |
|  | *VV1182* | VV1182 | ribonuclease T | ^a^ |
|  | *VV1223* | VV1223 | metal-dependent phosphoesterase | ^a^ |
|  | *VV1232* | VV1232 | DNA polymerase II | ^a^ |
|  | *VV1233* | VV1233 | Response regulator | ^a^ |
|  | *VV1264* | VV1264 | protein-tyrosine-phosphatase | |
|  | *VV1281* | VV1281 | DNA polymerase III subunit delta' | |
|  | *VV1294* | VV1294 | DNA polymerase III subunit alpha | ^a^ |
|  | *VV1295* | VV1295 | hypothetical protein | |
|  | *VV1298* | VV1298 | periplasmic protease | ^a^ |
|  | *VV1379* | VV1379 | alpha-galactosidase | |
|  | *VV1478* | VV1478 | oligoendopeptidase F | ^a^ |
|  | *VV1494* | VV1494 | di- and tripeptidase | |
|  | *VV1592* | VV1592 | Zn-dependent carboxypeptidase | |
|  | *VV1616* | VV1616 | intercellular adhesion protein A | ^a^ |
|  | *VV1617* | VV1617 | glycosyltransferase | ^a^ |
|  | *VV1620* | VV1620 | glycosyltransferase | |
|  | *VV1622* | VV1622 | ExoQ family protein | |
|  | *VV1624* | VV1624 | hypothetical protein | ^a^ |
|  | *VV1625* | VV1625 | glycosyltransferase | ^a^ |
|  | *VV1626* | VV1626 | glycosyltransferase | ^a^ |
|  | *VV1647* | VV1647 | carboxy-terminal protease | ^a^ |
|  | *VV1997* | VV1997 | chitinase | ^a^ |
|  | *VV2007* | VV2007 | Flp pilus assembly protein TadA | |
|  | *VV2025* | VV2025 | DnaK-like molecular chaperone | |
|  | *VV2026* | VV2026 | DnaK-like molecular chaperone | ^a^ |
|  | *VV2035* | VV2035 | glycosyltransferase protein | |
|  | *VV2036* | VV2036 | glycosyltransferase protein | |
|  | *VV2037* | VV2037 | glycosyltransferase protein | ^a^ |
|  | *VV2043* | VV2043 | capsular polysaccharide biosynthesis glycosyltransferase | ^a^ |
|  | *VV2101* | VV2101 | DNA polymerase III subunit epsilon | ^a^ |
|  | *VV2114* | VV2114 | hypothetical protein | |
|  | *VV2117* | VV2117 | lipoprotein |  |
|  | *VV2119* | VV2119 | iron-regulated protein | |
|  | *VV2353* | VV2353 | 3-deoxy-manno-octulosonate cytidylyltransferase | ^a^ |
|  | *VV2411* | VV2411 | DNA polymerase III subunits gamma and tau | |
|  | *VV2439* | VV2439 | peptidase insulinase family protein | ^a^ |
|  | *VV2442* | VV2442 | RNA polymerase sigma factor | |
|  | *VV2500* | VV2500 | succinyl-diaminopimelate desuccinylase | |
|  | *VV2508* | VV2508 | Zn-dependent protease | |
|  | *VV2529* | VV2529 | DNA polymerase III subunit epsilon | ^a^ |
|  | *VV2545* | VV2545 | UDP-N-acetylglucosamine acyltransferase | ^a^ |
|  | *VV2558* | VV2558 | methionine aminopeptidase | ^a^ |
|  | *VV2628* | VV2628 | acetyltransferase | |
|  | *VV2651* | VV2651 | Flp pilus assembly protein TadC | |
|  | *VV2652* | VV2652 | Flp pilus assembly protein TadB | ^a^ |
|  | *VV2653* | VV2653 | Flp pilus assembly protein TadA | ^a^ |
|  | *VV2654* | VV2654 | Flp pilus assembly protein, ATPase CpaE | |
|  | *VV2660* | VV2660 | Flp pilus assembly protein, secretin CpaC | ^a^ |
|  | *VV2661* | VV2661 | Flp pilus assembly protein CpaB | ^a^ |
|  | *VV2687* | VV2687 | DNA polymerase III subunit psi | |
|  | *VV2691* | VV2691 | collagenase | ^a^ |
|  | *VV2715* | VV2715 | ATP-dependent Zn protease | ^a^ |
|  | *VV2724* | VV2724 | membrane protein | |
|  | *VV2759* | VV2759 | DnaK suppressor protein | ^a^ |
|  | *VV2781* | VV2781 | type IV prepilin-like proteins leader peptide processing enzyme | |
|  | *VV2795* | VV2795 | Zn-dependent peptidase | ^a^ |
|  | *VV2808* | VV2808 | DNA-directed RNA polymerase, sigma subunit | ^a^ |
|  | *VV2810* | VV2810 | membrane protein | |
|  | *VV2832* | VV2832 | signal peptidase I | ^a^ |
|  | *VV2838* | VV2838 | RNA polymerase sigma factor RpoE | ^a^ |
|  | *VV2907* | VV2907 | leucyl aminopeptidase | ^a^ |
|  | *VV2908* | VV2908 | DNA polymerase III subunit chi | ^a^ |
|  | *VV2925* | VV2925 | ATP-dependent helicase HepA | ^a^ |
|  | *VV2990* | VV2990 | type IV pilus (Tfp) assembly protein PilQ | |
|  | *VV2996* | VV2996 | DNA-binding transcriptional regulator OxyR | |
|  | *VV3003* | VV3003 | acetylornithine deacetylase | ^a^ |
|  | *VV3008* | VV3008 | Cystathionine gamma_synthase | ^a^ |
|  | *VV3015* | VV3015 | ATP-dependent protease peptidase subunit | |
|  | *VV3074* | VV3074 | N-acetylmuramoyl-L-alanine amidase | ^a^ |
|  | *VV3090* | VV3090 | membrane-bound metallopeptidase | |
|  | *VV3132* | VV3132 | DNA polymerase III subunit epsilon | |
|  | *VV3145* | VV3145 | Bacterial nucleoid DNA | |
|  | *VV3156* | VV3156 | anti-RNA polymerase sigma 70 factor | |
|  | *VV3158* | VV3158 | DNA-directed RNA polymerase subunit beta' | ^a^ |
|  | *xni* | VV0883 | exonuclease IX | |
| ChroII Core Virulence genes | *murP* | VVA1668 | PTS system N-acetylmuramic acid transporter subunit IIBC | |
|  | *murQ* | VVA1667 | N-acetylmuramic acid 6-phosphate etherase | |
|  | *VVA0044* | VVA0044 | chitinase |  |
|  | *VVA0077* | VVA0077 | Glucan phosphorylase | |
|  | *VVA0205* | VVA0205 | collagenase | ^a^ |
|  | *VVA0236* | VVA0236 | peptidase | ^a^ |
|  | *VVA0289* | VVA0289 | hydrolase |  |
|  | *VVA0296* | VVA0296 | iron-regulated membrane protein | ^a^ |
|  | *VVA0302* | VVA0302 | serine protease | ^a^ |
|  | *VVA0344* | VVA0344 | hypothetical protein | ^a^ |
|  | *VVA0361* | VVA0361 | oligopeptidase B | ^a^ |
|  | *VVA0362* | VVA0362 | heme transport protein | ^a^ |
|  | *VVA0363* | VVA0363 | hypothetical protein | ^a^ |
|  | *VVA0404* | VVA0404 | hypothetical protein | ^a^ |
|  | *VVA0424* | VVA0424 | periplasmic protein TonB1 | |
|  | *VVA0444* | VVA0444 | hypothetical protein | ^a^ |
|  | *VVA0517* | VVA0517 | hypothetical protein | ^a^ |
|  | *VVA0524* | VVA0524 | hypothetical protein | ^a^ |
|  | *VVA0537* | VVA0537 | Zinc metalloprotease | |
|  | *VVA0557* | VVA0557 | deacylase | ^a^ |
|  | *VVA0593* | VVA0593 | rough colony protein RcpA | |
|  | *VVA0595* | VVA0595 | hypothetical protein | ^a^ |
|  | *VVA0596* | VVA0596 | TadA protein | ^a^ |
|  | *VVA0597* | VVA0597 | Flp pilus assembly protein TadB | ^a^ |
|  | *VVA0599* | VVA0599 | Flp pilus assembly protein TadD | |
|  | *VVA0649* | VVA0649 | response regulator | ^a^ |
|  | *VVA0720* | VVA0720 | chitinase | ^a^ |
|  | *VVA0754* | VVA0754 | Zn-dependent protease | |
|  | *VVA0755* | VVA0755 | Zn-dependent protease with chaperone function | ^a^ |
|  | *VVA0781* | VVA0781 | heme receptor | |
|  | *VVA0782* | VVA0782 | transcriptional regulator | |
|  | *VVA0916* | VVA0916 | periplasmic protein TonB2 | ^a^ |
|  | *VVA0929* | VVA0929 | hypothetical protein | ^a^ |
|  | *VVA0953* | VVA0953 | dienelactone hydrolase | ^a^ |
|  | *VVA0965* | VVA0965 | cytotoxin, cytolysin VvhA | ^a^ |
|  | *VVA1034* | VVA1034 | RTX toxin transporter | |
|  | *VVA1036* | VVA1036 | RTX toxin secretion ATP-binding protein | |
|  | *VVA1081* | VVA1081 | peptidase T | ^a^ |
|  | *VVA1208* | VVA1208 | poly(3-hydroxyalkanoate) synthetase | |
|  | *VVA1303* | VVA1303 | vulnibactin utilization protein | ^a^ |
|  | *VVA1372* | VVA1372 | carboxypeptidase G2 | ^a^ |
|  | *VVA1405* | VVA1405 | DNA polymerase III subunit epsilon | |
|  | *VVA1612* | VVA1612 | multidrug ABC transporter ATPase and permease | ^a^ |
|  | *VVA1654* | VVA1654 | toxin secretion ABC transporter ATP-binding subunit | ^a^ |
|  | *VVA1672* | VVA1672 | exoprotease Vcc | ^a^ |
|  | *VVA1680* | VVA1680 | polymerase |  |
| ChroI Non Core Virulence genes | *depG* | VV0591 | protease DO | |
|  | *flgE* | VV0963 | flagellar hook protein FlgE | |
|  | *fur* | VV1014 | ferric uptake regulator | |
|  | *nagA* | VV1011 | N-acetylglucosamine-6-phosphate deacetylase | |
|  | *pepN* | VV1650 | aminopeptidase N | |
|  | *SmcR* | VV2770 | SmcR-like protein VvpR | |
|  | *tldE* | VV0441 | peptidase PmbA | |
|  | *VV0052* | VV0052 | RNA polymerase ECF-type sigma factor | |
|  | *VV0063* | VV0063 | hypothetical protein | |
|  | *VV0166* | VV0166 | outer membrane cobalamin receptor protein | |
|  | *VV0281* | VV0281 | lipid A biosynthesis lauroyl acyltransferase | |
|  | *VV0294* | VV0294 | lipopolysaccharide biosynthesis glycosyltransferase | |
|  | *VV0297* | VV0297 | 3-deoxy-D-manno-octulosonic-acid kinase | |
|  | *VV0299* | VV0299 | glycosyl transferase family protein | |
|  | *VV0301* | VV0301 | D-glucose-1-phosphate thymidylyltransferase | |
|  | *VV0302* | VV0302 | dTDP-4-dehydrorhamnose reductase | |
|  | *VV0303* | VV0303 | dTDP-6-deoxy-D-xylo-4-hexulose-3,5-epimerase | |
|  | *VV0309* | VV0309 | nucleoside-diphosphate sugar epimerase | |
|  | *VV0311* | VV0311 | UDP-N-acetylglucosamine 2-epimerase | |
|  | *VV0324* | VV0324 | 3-deoxy-D-manno-octulosonic-acid transferase | |
|  | *VV0326* | VV0326 | lipid A biosynthesis (KDO)2-(lauroyl)-lipid IVA acyltransferase | |
|  | *VV0337* | VV0337 | outer membrane capsular polysaccharide transport protein | |
|  | *VV0340* | VV0340 | tyrosine-protein kinase Wzc | |
|  | *VV0341* | VV0341 | UDP-N-acetylglucosamine 2-epimerase | |
|  | *VV0345* | VV0345 | hypothetical protein | |
|  | *VV0346* | VV0346 | hypothetical protein | |
|  | *VV0347* | VV0347 | hypothetical protein | |
|  | *VV0348* | VV0348 | hypothetical protein | |
|  | *VV0360* | VV0360 | glycosyltransferase | |
|  | *VV0364* | VV0364 | nucleoside-diphosphate sugar epimerase | |
|  | *VV0369* | VV0369 | transposase |  |
|  | *VV0545* | VV0545 | transposase |  |
|  | *VV0627* | VV0627 | hypothetical protein | |
|  | *VV0825* | VV0825 | heat shock protein GrpE | |
|  | *VV1049* | VV1049 | hypothetical protein | |
|  | *VV1075* | VV1075 | acyltransferase | |
|  | *VV1771* | VV1771 | retron-type reverse transcriptase | |
|  | *VV1999* | VV1999 | hypothetical protein | |
|  | *VV2001* | VV2001 | hypothetical protein | |
|  | *VV2003* | VV2003 | Flp pilus assembly protein TadD | |
|  | *VV2005* | VV2005 | Flp pilus assembly protein TadC | |
|  | *VV2006* | VV2006 | Flp pilus assembly protein TadB | |
|  | *VV2008* | VV2008 | Flp pilus assembly protein CpaE-like | |
|  | *VV2010* | VV2010 | Flp pilus assembly protein, secretin CpaC | |
|  | *VV2011* | VV2011 | Flp pilus assembly protein CpaB | |
|  | *VV2012* | VV2012 | hypothetical protein | |
|  | *VV2200* | VV2200 | DNA or RNA helicase | |
|  | *VV2335* | VV2335 | hypothetical protein | |
|  | *VV2357* | VV2357 | lipid transporter ATP-binding/permease | |
|  | *VV2457* | VV2457 | Chemotaxis signal transduction protein | |
|  | *VV2581* | VV2581 | DNA polymerase IV | |
|  | *VV2599* | VV2599 | RNA polymerase sigma factor | |
|  | *VV2658* | VV2658 | hypothetical protein | |
|  | *VV2761* | VV2761 | polyA polymerase | |
|  | *VV2778* | VV2778 | type IV pilin PilA | |
|  | *VV2911* | VV2911 | Zn-dependent protease with chaperone function | |
|  | *VV2973* | VV2973 | zinc uptake regulation protein | |
|  | *ychF* | VV0924 | GTP-dependent nucleic acid-binding protein EngD | |
| ChroI Non Core Virulence genes | *VVA0045* | VVA0045 | chitinase |  |
|  | *VVA0085* | VVA0085 | pyrrolidone-carboxylate peptidase | |
|  | *VVA0098* | VVA0098 | Zn-dependent peptidase | |
|  | *VVA0166* | VVA0166 | alpha-galactosidase | |
|  | *VVA0332* | VVA0332 | RTX toxin secretion ATP-binding protein | |
|  | *VVA0384* | VVA0384 | calcium-binding protein | |
|  | *VVA0389* | VVA0389 | glycosyltransferase | |
|  | *VVA0391* | VVA0391 | lipopolysaccharide biosynthesis protein | |
|  | *VVA0395* | VVA0395 | capsular polysaccharide biosynthesis glycosyltransferase | |
|  | *VVA0439* | VVA0439 | hypothetical protein | |
|  | *VVA0449* | VVA0449 | Signal transduction histidine kinase | |
|  | *VVA0504* | VVA0504 | hypothetical protein | |
|  | *VVA0590* | VVA0590 | hypothetical protein | |
|  | *VVA0598* | VVA0598 | Flp pilus assembly protein TadC | |
|  | *VVA0781* | VVA0781 | heme receptor | |
|  | *VVA0813* | VVA0813 | hypothetical protein | |
|  | *VVA0866* | VVA0866 | hypothetical protein | |
|  | *VVA0964* | VVA0964 | cytolysin secretion protein VvhB | |
|  | *VVA0973* | VVA0973 | Zn-dependent protease with chaperone function | |
|  | *VVA1030* | VVA1030 | RTX repeat-containing cytotoxin | |
|  | *VVA1259* | VVA1259 | serine protease | |
|  | *VVA1309* | VVA1309 | ferric vulnibactin outer membrane receptor | |
|  | *VVA1337* | VVA1337 | TagA-related protein | |
|  | *VVA1405* | VVA1405 | DNA polymerase III subunit epsilon | |
|  | *VVA1436* | VVA1436 | DNA-binding protein | |
|  | *VVA1465* | VVA1465 | Zinc metalloprotease, *Vibrio*lysin | |
|  | *VVA1495* | VVA1495 | DNA polymerase III subunit alpha | |
|  | *VVA1508* | VVA1508 | ferric aerobactin receptor | |
|  | *VVA1510* | VVA1510 | N-acetylglucosamine-6-phosphate deacetylase | |
|  | *VVA1544* | VVA1544 | hypothetical protein | |
|  | *VVA1677* | VVA1677 | toxin secretion ABC transporter ATP-binding subunit | |
